# The relationship between gut microbiota and neurodegenerative diseases: a genetic and epigenetic perspective

**DOI:** 10.1007/s11011-026-01798-9

**Published:** 2026-02-28

**Authors:** Zeynep Betül Altan, Murat Ihlamur

**Affiliations:** 1 Department of Biotechnology, Institute of Health Sciences, Bezmialem Vakif University, Istanbul, Türkiye; 2 Department of Bioengineering, Faculty of Chemical and Metallurgical Engineering, Yildiz Technical University, Istanbul, Türkiye; 3 Department of Electronics and Automation, Vocational School, Biruni University, Istanbul, Türkiye

**Keywords:** Intestinal microbiota, Neurodegenerative diseases, Genetic and epigenetic mechanisms, Gut-brain axis

## Abstract

Gut microbiota (GM) is a complex and dynamic structure that can have a wide range of effects on human health. Studies in recent years have shown that microbiota is not only related to the gastrointestinal tract (GIT) but also to the immune and endocrine systems. It causes various effects on host physiology, especially through genetic and epigenetic mechanisms. It shows that microbiota-derived metabolites can play a role in the development of neurological diseases by changing gene expression. In this review article, the relationship between GM and neurodegenerative diseases (NDs) is explained in terms of genetics and epigenetics. In terms of the gut–brain axis (GBA); the role of systems such as short-chain fatty acids (SCFA), vagus nerve, inflammatory responses and intestinal permeability in the pathogenesis of NDs such as Alzheimer's (AD), Parkinson's (PD), Huntington's (HD) and Multiple Sclerosis (MS) is discussed. In addition, experimental studies have drawn attention to the effects of changes in microbiota on neuroinflammation and cognitive impairment. In terms of treatment strategies; Probiotics, prebiotics, synbiotics, fecal microbiota transplantation (FMT) and nutritional interventions targeting microbiota seem to be promising strategies. However, the fact that microbiota structure varies between individuals indicates that studies in this area should be conducted in a more personalized manner. This article aims to provide a basis for future research by approaching the relationship between microbiota and NDs in a holistic manner.

**Graphical Abstract:**

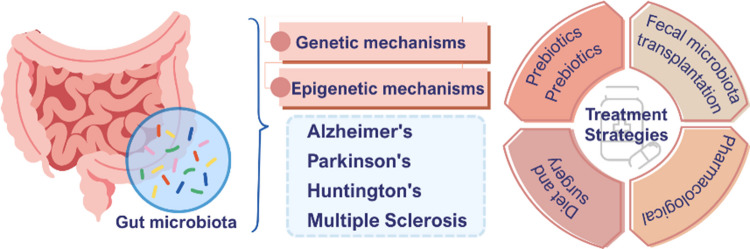

## Introduction

The human body comprises not only its own cells but also millions of colonizing microorganisms, together forming a highly complex biological system. These microorganisms include diverse taxa such as archaea, viruses, bacteria, and fungi. Recent evidence has confirmed that the number of microbial cells in the human body is approximately equivalent to the number of human cells (Li et al. 2016). A substantial proportion of this microbial community resides in the gastrointestinal tract (GIT), particularly in the intestines. The collective population of microorganisms inhabiting the intestines is referred to as the “gut microbiota (GM)” (Clemente et al. 2012).

The GM represents a dynamic and diverse microbial ecosystem living in symbiosis with the host and undertaking critical physiological functions. These include supporting digestion, modulating immunity, synthesizing essential vitamins, and protecting the host against pathogenic microorganisms (Gomaa 2020; Sommer and Bäckhed 2013). Importantly, the influence of the GM extends beyond the GIT, with compelling evidence linking it to metabolism, immune regulation, behavioral processes, and central nervous system (CNS) functions (Mu et al. 2016). The bidirectional communication between the GM and CNS is mediated through neural, hormonal, metabolic, and immunological pathways, collectively referred to as the gut–brain axis (GBA) (Quigley 2017).

The activity of the GBA involves multiple mechanisms, including the hypothalamic–pituitary–adrenal (HPA) axis, the vagus nerve, the intestinal epithelium, and small signaling molecules such as short-chain fatty acids (SCFAs). Recent studies have emphasized not only the physiological but also the genetic and epigenetic dimensions of GM function. For instance, metabolites produced by the microbiota—particularly SCFAs—have been shown to modulate epigenetic mechanisms, leading to significant alterations in gene expression (Fan et al. 2024). Conversely, host genetic variants can shape microbial composition, underscoring the reciprocal nature of this relationship (Cuevas-Sierra et al. 2019). This interplay highlights the necessity of considering genetic susceptibility, epigenetic modifications, and microbial environmental factors as an integrated network.

This network of interactions is of particular significance in the context of neurodegenerative diseases (NDs). Alzheimer’s disease (AD), Parkinson’s disease (PD), Huntington’s disease (HD), and Multiple Sclerosis (MS) represent major NDs characterized by progressive neuronal injury and cell death, ultimately resulting in severe functional impairment. Several factors—including increased intestinal permeability, microbial dysbiosis, systemic inflammation, and neuroinflammation—are thought to contribute to their etiopathogenesis (Dandamudi et al. 2024; Fu et al. 2024; Zhu et al. 2022). Consequently, advancing our understanding of GM–NDs interactions is essential both for the identification of early-stage biomarkers and for the development of personalized microbiota-targeted therapeutic strategies.

In this review, we provide a comprehensive overview of the definition, structure, development, and functions of the GM, its links to genetic and epigenetic mechanisms, its contribution to the onset of NDs, and potential gut-targeted therapeutic approaches.

Beyond isolated descriptions of genetics, epigenetics, or gut microbiota alterations, emerging evidence suggests that neurodegenerative diseases (NDs) arise from a dynamic and bidirectional interplay between host genetic susceptibility, microbiota-derived signals, and epigenetic regulation. Host genetic background influences immune signaling, intestinal barrier integrity, and metabolic pathways that collectively shape gut microbial composition (Kurilshikov et al. 2017). In turn, gut microbiota–derived metabolites, including short-chain fatty acids, bile acid derivatives, and tryptophan metabolites, can modulate epigenetic mechanisms such as histone acetylation, DNA methylation, and microRNA expression, thereby influencing neuroinflammation, microglial activation, and blood–brain barrier function. These epigenetically mediated pathways provide a mechanistic link through which peripheral microbial signals may affect central nervous system homeostasis. Importantly, this gene–microbiota–epigenome axis does not operate uniformly across neurodegenerative disorders; rather, disease-specific molecular vulnerabilities determine which nodes of this network are most prominently affected. Understanding NDs through this integrated framework may help reconcile heterogeneous findings across studies and support the development of targeted, mechanism-driven microbiome-based interventions (Woo and Alenghat 2022).

Neurodegeneration is increasingly recognized as a systems-level disorder involving interactions across genetic, epigenetic, immunological, and metabolic domains. Within this context, the gut microbiome functions as a critical environmental interface that translates host genetic predisposition into molecular signals capable of influencing brain pathology. Genetic variants affecting immune regulation, mucosal defense, and metabolic homeostasis can predispose individuals to distinct microbial configurations, thereby shaping baseline inflammatory tone and metabolite availability (Kurilshikov et al. 2017).

Microbiota-derived metabolites act as key molecular intermediaries in this axis. Several of these compounds have been shown, primarily in preclinical models, to influence epigenetic regulators such as histone deacetylases, DNA methyltransferases, and non-coding RNAs. Through these mechanisms, microbial signals may modulate gene expression programs involved in microglial activation, cytokine production, synaptic plasticity, and neuronal survival (Erny and Prinz 2020).

Importantly, the downstream consequences of this gene–microbiome–epigenome interaction appear to be disease-specific. In Alzheimer’s disease, epigenetic modulation of inflammatory and amyloid-related pathways may be particularly relevant, whereas in Parkinson’s disease, enteric nervous system signaling and α-synuclein–associated immune responses emerge as dominant nodes. In multiple sclerosis, immune cell differentiation and peripheral tolerance are central targets, while in Huntington’s disease, mutant protein–driven cellular vulnerability may interact with microbiota-induced metabolic and inflammatory stressors (Chen et al. 2025).

Framing neurodegenerative disorders within this integrated triad provides a conceptual basis for interpreting inconsistent findings across studies and highlights why microbiome-targeted interventions may yield variable outcomes across diseases and individuals. This framework also underscores the need for mechanistically informed, disease-specific, and genetically stratified approaches when translating microbiome research into clinical applications (Kashyap et al. 2017).

## Methods and literature search strategy

A comprehensive literature search was conducted in PubMed, Scopus, Web of Science, and Google Scholar databases. The search included studies published between 2010 and 2024 using the following keywords: gut microbiota (GM), intestinal microbiota, NDs, Alzheimer’s disease, Parkinson’s disease, Huntington’s disease, MS, genetics, epigenetics, gut–brain axis. Both original research articles and review papers were considered. Wherever possible, priority was given to primary research articles (clinical, in vivo, and mechanistic studies), while review papers were primarily used to provide background context and to identify additional primary sources. Inclusion criteria were: (i) studies published in English, (ii) studies focusing on the role of GM in NDs, and (iii) articles addressing genetic or epigenetic mechanisms. Exclusion criteria were: (i) non-peer-reviewed articles, (ii) conference abstracts without full text, and (iii) studies unrelated to GM or neurodegeneration. This review was designed as a narrative synthesis rather than a formal systematic review. Therefore, no quantitative meta-analysis or standardized risk-of-bias assessment was performed. However, to enhance transparency, we prioritized peer-reviewed articles in high-impact journals and critically evaluated study design, sample size, and relevance to the gut–brain axis. Both preclinical (in vitro and animal) and clinical (observational and interventional) studies were considered. Reference lists of key publications were also screened to identify additional relevant articles that were not captured in the initial database searches.

To improve interpretability and consistency, the included studies were qualitatively categorized according to study type (clinical, animal, or in vitro), disease focus, and primary mechanistic emphasis (genetic, epigenetic, immunological, or metabolic). Greater weight was given to human studies, longitudinal designs, and investigations integrating host and microbiome-related data. Preclinical studies were primarily used to support mechanistic plausibility rather than direct clinical inference. This structured narrative approach was adopted to balance comprehensive coverage with critical evaluation, acknowledging the inherent heterogeneity of microbiome research in neurodegenerative diseases.

### Gut microbiota: definition, development, and functions

The gut microbiota (GM) is a dynamic and complex community of microorganisms residing in the human GIT and living in a symbiotic relationship with the host. The term microbiota broadly encompasses a wide range of microorganisms, including viruses, fungi, bacteria, archaea, and protozoa. These organisms have the potential to influence various aspects of human physiology (Gomaa 2020). The collective genetic material of these microorganisms, together with their environmental interactions, constitutes what is referred to as the microbiota (Clemente et al. 2012).

From an evolutionary perspective, the human GM has co-developed alongside its host. As the host organism matures, the microbiota shapes, and is shaped by, intestinal development at both morphological and immunological levels (Sommer and Bäckhed 2013). Throughout evolution, the microbiota has emerged not only as a determinant of digestive function but also as a fundamental factor influencing host physiology and adaptive capacity (Cunningham et al. 2021).

The embryonic intestine is sterile, but colonization begins immediately after birth through exposure to environmental microorganisms. Factors such as mode of delivery, medication use before and during birth, breastfeeding, environmental conditions, and host genetics play critical roles in shaping this colonization process (Li et al. 2016). Once established, the microbiota remains dynamic, undergoing modifications throughout life in response to lifestyle and environmental influences. Early-life nutrition and exposure to external environments are particularly important, as they affect both the composition and functional capacity of the GM (Cuevas-Sierra et al. 2019).

In healthy individuals, the GM comprises a broad diversity of bacterial species, with Bacteroidetes, Firmicutes, and Proteobacteria being the most prominent phyla (Clemente et al. 2012). Variability in GM composition among individuals is largely attributable to diet, age, genetic background, medication use, and lifestyle, which together contribute to the unique development of each person’s microbiota (Baothman et al. 2016). This individuality highlights the potential of the microbiota to serve as a biomarker of health status.

One of the most critical physiological contributions of the GM is the production of short-chain fatty acids (SCFAs). SCFAs such as butyrate, propionate, and acetate provide energy to intestinal epithelial cells, help preserve intestinal barrier integrity, and reduce inflammation (Flint et al. 2012). Butyrate, in particular, not only fuels epithelial cells but also exerts anti-inflammatory effects and plays central roles in immune regulation and epigenetic modifications (Cuevas-Sierra et al. 2019).

Beyond digestion, the GM is also involved in the production of neurotransmitters, thereby contributing to neural communication. It has been shown to facilitate the synthesis of precursors for neurotransmitters such as serotonin, gamma-aminobutyric acid (GABA), and dopamine (Mu et al. 2016). These metabolites are essential for proper central nervous system (CNS) function and can influence behavioral processes, thereby supporting the link between GM composition and neurological or psychiatric disorders (Sittipo et al. 2022; Xu and Lu 2025).

In addition, the GM plays a vital role in host defense. Beneficial bacteria inhibit the colonization of pathogenic microorganisms by competing for physical niches and suppressing the production of harmful metabolites (Gomaa 2020). The GM also supports mucosal immunity by contributing to immune homeostasis. This is mediated through mechanisms such as the neonatal immune response, activation of regulatory T (Treg) cells, and cytokine production (Li et al. 2016).

**Fig. 1:**
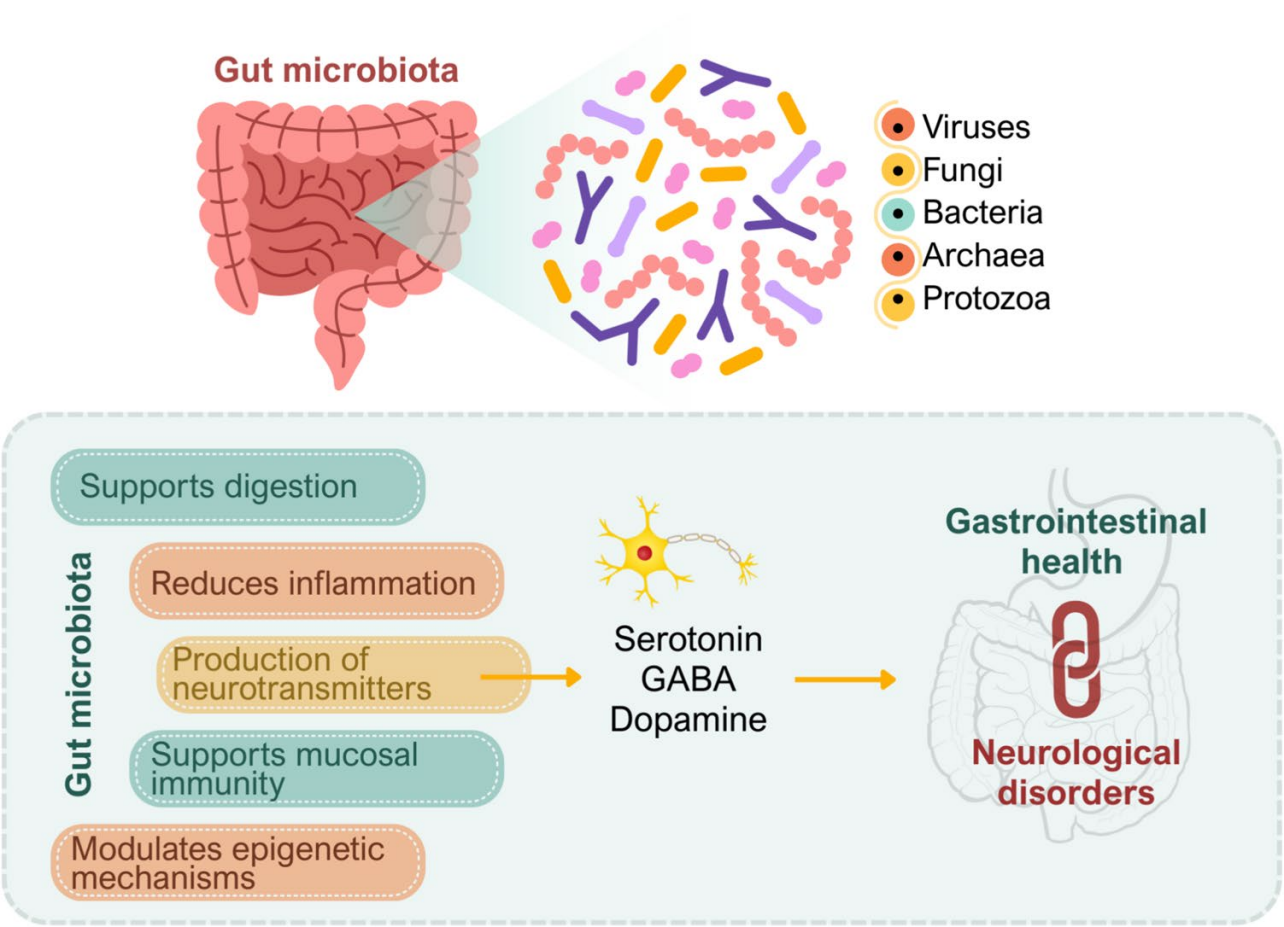
Schematic overview of the integrated interactions between host genetics, gut microbiota, and epigenetic regulation. Host genetic susceptibility influences immune signaling, intestinal barrier integrity, and metabolic pathways that collectively shape gut microbial composition. Microbiota-derived metabolites, including short-chain fatty acids, bile acid derivatives, and tryptophan metabolites, modulate epigenetic mechanisms such as histone acetylation, DNA methylation, and non-coding RNA expression. These epigenetic modifications influence neuroinflammation, microglial activation, blood–brain barrier integrity, and downstream neurodegenerative processes

Collectively, these functions illustrate that the GM regulates not only gastrointestinal health but also the well-being of other organ systems, particularly the nervous system. Thus, it should be regarded as a multifaceted component essential for maintaining overall human health (Yarandi et al. 2016). An overview of the composition and key functions of the GM, and its links to gastrointestinal and neurological health, is illustrated in Fig. 1.

### Interaction with genetic and epigenetic mechanisms

GM plays a critical role not only in physiological processes such as immunity and digestion but also through its interactions with the host’s genetic and epigenetic architecture. This bidirectional relationship operates as a dynamic balance in which host genetics influence microbiota composition, while the microbiota in turn modulates host gene expression (Goodrich et al. 2016a, b).

Host genetic variability is a major contributor to interindividual differences in gut microbiota composition, function, and ecological stability, and therefore represents a key determinant of why microbiome–disease associations are often heterogeneous across cohorts (Goodrich et al. 2016a, b). Genetic variation can influence microbial colonization patterns through several convergent host pathways, including (i) innate and adaptive immune responsiveness, (ii) mucosal barrier integrity and mucus glycosylation, and (iii) metabolic features that shape nutrient availability in the gut lumen. These host-driven factors alter the selective pressures within the intestinal ecosystem, thereby affecting microbial diversity, metabolite production capacity, and resilience to external perturbations such as diet, antibiotics, and inflammation. Importantly, the same microbial exposure may lead to distinct immune–metabolic outcomes in different individuals depending on their genetic background, which can ultimately contribute to differential susceptibility to chronic inflammation and downstream neurodegenerative processes. Thus, genetic stratification and consideration of host variability are essential for interpreting microbiome signatures and for developing precision microbiome interventions that are both disease- and patient-specific (Kurilshikov et al. 2017).

The interaction between host genetics and the GM begins with the observation that specific genetic variants can directly shape microbial diversity and composition. This finding suggests that interindividual microbial differences are driven not only by environmental factors but also by genetic determinants (Celiker and Kalkan 2020). While genetic architecture can dictate bacterial diversity within the GM, the microbiota reciprocally influences host genetic regulation and epigenetic programming. Studies have shown that genetic variations in immune-related genes may promote the dominance of specific microbial taxa, thereby altering microbial balance. Such genetic effects can modify competitive interactions among microbial populations, enabling certain species to thrive while others decline (Cuevas-Sierra et al. 2019). This genetic–microbiota interplay directly affects physiological processes including immune responses, inflammatory regulation, and mucosal defense. Moreover, host genetic effects on microbiota may interact with epigenetic plasticity, meaning that environmentally induced epigenetic changes can amplify or buffer genetically predisposed immune and barrier phenotypes. This layered interaction provides a plausible explanation for variable clinical trajectories and inconsistent microbiome signatures reported across neurodegenerative disease cohorts. For example, altered expression of immune-regulatory genes may disrupt microbial balance, thereby amplifying inflammatory responses—a mechanism that may underlie the development of autoimmune and other chronic diseases (Montgomery et al. 2020).

Beyond genetic interactions, the GM exerts substantial influence on epigenetic mechanisms. Epigenetic regulation involves heritable modifications in gene expression that occur without altering the underlying DNA sequence. The principal epigenetic regulators include histone modifications, DNA methylation, and small non-coding RNAs. These mechanisms provide a key link between environmental exposures and long-term host physiological outcomes (Fan et al. 2024). Microbial metabolites, particularly short-chain fatty acids (SCFAs), directly modulate these epigenetic regulators. SCFAs alter gene expression in immune and intestinal epithelial cells, thereby exerting broad effects on host physiology (Lin et al. 2015).

Butyrate, in particular, functions as a histone deacetylase (HDAC) inhibitor, thereby increasing histone acetylation. Enhanced acetylation relaxes chromatin structure, facilitating transcriptional activation of specific genes. Consequently, some genes exhibit upregulated expression while others are suppressed, leading to systemic effects on immune responses, inflammatory pathways, and cellular differentiation (Fan et al. 2024). Experimental evidence has shown that butyrate inhibits HDAC activity in human colonic cells by approximately 30%, resulting in a ~ 40% increase in histone H3 acetylation. Similarly, SCFAs have been shown to affect DNA methylation, reducing promoter-region methylation by ~ 15–20% in certain cell lines (Krautkramer et al. 2021). These findings demonstrate that SCFAs not only exert structural but also functional influence at the level of gene regulation.

The epigenetic effects of the microbiota extend to the central nervous system (CNS). Microbial metabolites and cell wall components have been reported to regulate the expression of neurodevelopment-related genes, potentially increasing susceptibility to neurodegenerative and neuropsychiatric disorders (Nohesara et al. 2023). This evidence highlights the possibility that microbiota composition during early developmental stages—childhood and adolescence—may exert long-lasting effects on neurological health. Furthermore, as epigenetic modifications are shaped by environmental conditions and stabilized during early life, they may contribute not only to heightened disease susceptibility but also to the transgenerational reshaping of genetic expression under environmental influence (Celiker and Kalkan 2020).

### The gut–brain axis and the role of the microbiota in neurodegenerative diseases

The gut–brain axis (GBA) constitutes a bidirectional communication network linking the enteric nervous system (ENS), the central nervous system (CNS), and the GM. This axis operates through hormonal, immunological, neural, and metabolic pathways, thereby maintaining systemic homeostasis (Mu et al. 2016). Key elements contributing to the functionality of the GBA include the vagus nerve, the hypothalamic–pituitary–adrenal (HPA) axis, the intestinal epithelial barrier, and metabolites produced by the GM (Quigley 2017). By regulating the synthesis of neuroactive compounds, the GM can directly or indirectly influence brain function. Microbiota-derived short-chain fatty acids (SCFAs), along with neurotransmitters such as dopamine, serotonin, and gamma-aminobutyric acid (GABA), play pivotal roles in this process (Mu et al. 2016). SCFAs also support the integrity of the blood–brain barrier (BBB), thereby reducing neuroinflammation (Hirschberg et al. 2019).

Beyond a simple bidirectional communication pathway, the gut–brain axis represents a complex, multi-system network integrating neural, immune, endocrine, and metabolic signaling routes. Neural communication is mediated primarily through the enteric nervous system and the vagus nerve, enabling rapid transmission of microbial and intestinal signals to central autonomic and limbic brain regions. In parallel, immune pathways link gut microbiota to brain function via cytokine release, microglial priming, and peripheral immune cell trafficking, thereby influencing neuroinflammatory tone (Carabotti et al. 2015).

**Fig. 2:**
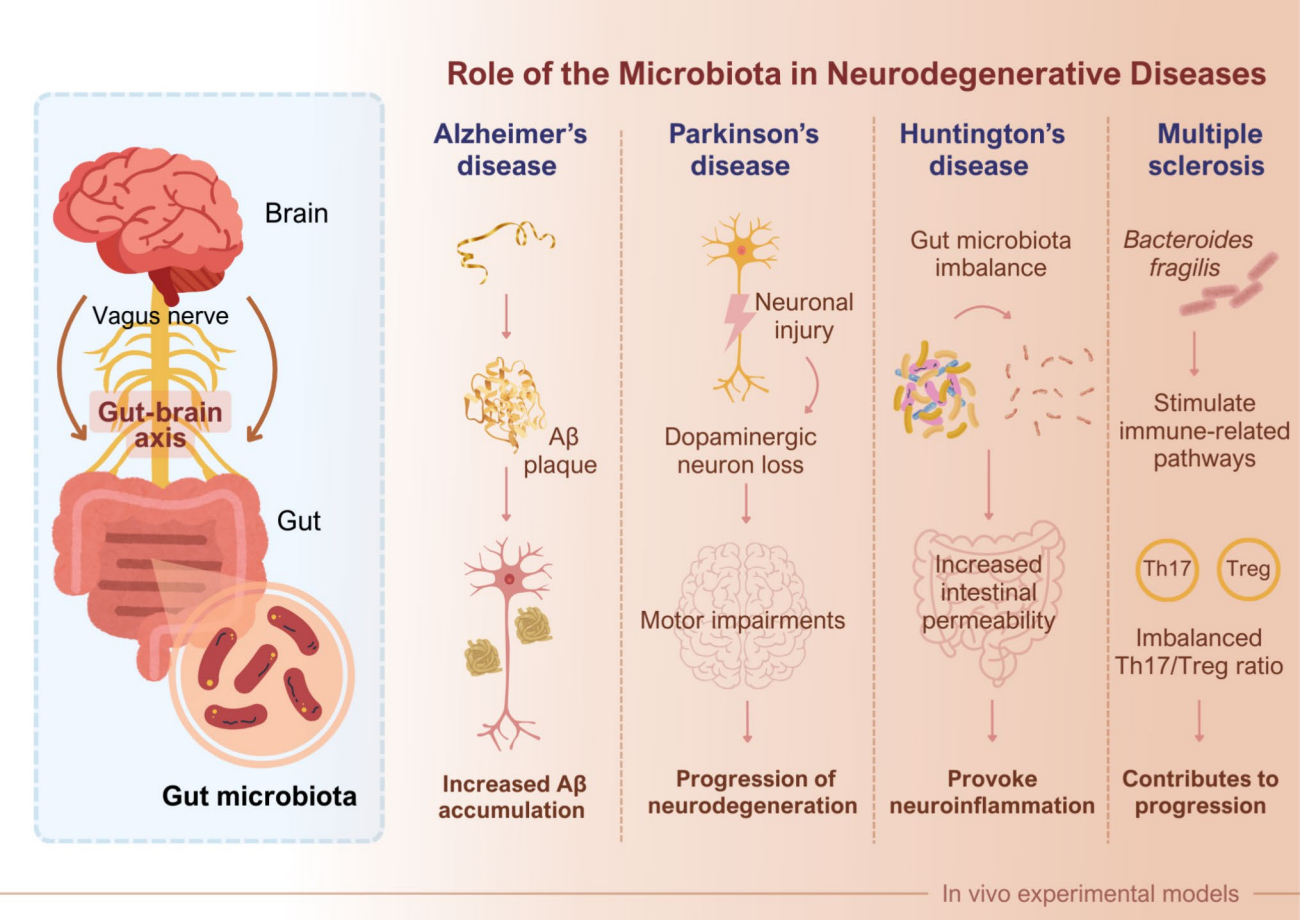
Role of the gut microbiota in neurodegenerative diseases

Endocrine signaling further contributes to gut–brain communication through modulation of the hypothalamic–pituitary–adrenal axis, stress hormone release, and neuroendocrine feedback loops that can, in turn, reshape gut microbial composition. Metabolic pathways constitute an additional layer of integration, as microbiota-derived metabolites such as short-chain fatty acids, bile acid derivatives, and tryptophan metabolites act as systemic signaling molecules capable of influencing blood–brain barrier integrity, epigenetic regulation, and neuronal function. Importantly, these neural, immune, endocrine, and metabolic components do not operate in isolation but form an interconnected regulatory network, through which alterations in gut microbial ecology may exert pleiotropic effects on central nervous system homeostasis and vulnerability to neurodegenerative processes (Woo and Alenghat 2022). These disease-specific and shared microbiota-driven mechanisms along the gut–brain axis are summarized in Fig. 2. Specifically, the right panel highlights how dysbiosis-driven inflammatory cues and metabolite shifts (e.g., reduced SCFAs) can converge on barrier dysfunction and neuroinflammation in a disease-specific manner.

The left panel depicts the bidirectional communication between the GM and the brain via the vagus nerve, forming the gut–brain axis. The right panel summarizes microbiota-related mechanisms implicated in major neurodegenerative diseases. In Alzheimer’s disease, gut dysbiosis is associated with increased intestinal permeability and systemic inflammation, and microbiota-derived metabolites may modulate neuroinflammatory and Aβ-related pathways, contributing to plaque burden in preclinical models. In Parkinson’s disease, dysbiosis-associated endotoxins and altered microbial metabolites (e.g., reduced SCFA availability) may amplify immune activation and barrier dysfunction, facilitating neuroinflammatory signaling that contributes to dopaminergic neuron vulnerability and motor impairments. In Huntington’s disease, GM imbalance and increased intestinal permeability provoke neuroinflammation. In MS, specific bacterial taxa (such as Bacteroides fragilis) stimulate immune-related pathways and disturb the Th17/Treg balance, contributing to disease progression.

From a pathophysiological perspective, the contribution of the gut microbiota to neurodegenerative diseases can be conceptualized across three partially overlapping stages: initiation, disease amplification, and progression (Ryman et al. 2023). In the initiation stage, microbiota-related factors may increase vulnerability through chronic low-grade inflammation, impaired intestinal barrier integrity, altered immune priming, and changes in neuroactive and epigenetically active metabolites. During disease amplification, gut-derived inflammatory mediators and metabolic signals can reinforce neuroinflammatory cascades, microglial activation, and blood–brain barrier dysfunction, thereby potentiating disease-relevant molecular pathways (e.g., protein misfolding and aggregation, synaptic impairment). In the progression stage, microbiota alterations may further modulate symptom severity and trajectory through sustained immune–metabolic dysregulation, medication–microbiome interactions, and reduced resilience of microbial ecosystems. Importantly, the relative contribution of these stages may differ by disorder (AD, PD, MS, HD) and by individual patient characteristics, which provides a mechanistic explanation for heterogeneous microbiome signatures and variable therapeutic responses (Loh et al. 2024).

The vagus nerve provides a direct signaling route between the gut and the brain and is highly sensitive to alterations in microbial composition. Microbial components and metabolites activate vagal afferent fibers, transmitting signals to the CNS (Quigley 2017). These signals influence emotional states, cognitive function, and anxiety-related behaviors. Disruptions in the microbiota may increase systemic inflammation and intestinal permeability, a phenomenon often referred to as “leaky gut.” Elevated proinflammatory cytokines entering circulation can activate microglia within the brain, thereby contributing to neuroinflammation (Hirschberg et al. 2019).

### Alzheimer’s disease

The role of the GM in Alzheimer’s disease (AD) has been extensively investigated in experimental models. Studies in germ-free (GF) mice have shown reduced β-amyloid (Aβ) accumulation compared to conventionally colonized mice, suggesting that the microbiota contributes to Aβ plaque formation (Sun et al. 2021). Furthermore, fecal microbiota transplantation (FMT) from AD patients into healthy mice has resulted in cognitive impairments and increased plaque deposition in the recipient animals (Zhu et al. 2022). Similar findings have shown that dysbiotic mice exhibit higher levels of Aβ plaque formation, whereas mice with healthy microbiota display reduced accumulation (Sun et al. 2021). Transplantation of the microbiota from 16-month-old APP/PS1 transgenic mice into 3-month-old recipients significantly increased Aβ deposition in the latter, while FMT from AD patients likewise impaired cognition and enhanced plaque burden in healthy mice (Zhu et al. 2022). Collectively, these studies indicate that the GM contributes not only to neuroinflammation but also directly to pathological Aβ formation (Sun et al. 2021).

Despite growing evidence linking gut microbiota alterations to Alzheimer’s disease, the current literature remains heterogeneous and largely associative. While several studies consistently report reduced abundance of short-chain fatty acid–producing taxa and increased pro-inflammatory microbial signatures in AD cohorts, other investigations fail to detect robust disease-specific microbiota patterns, particularly after adjustment for age, diet, medication use, and gastrointestinal comorbidities. These discrepancies likely reflect methodological variability, including differences in sequencing platforms (16S rRNA vs. shotgun metagenomics), cohort size, disease stage, and control of confounding factors such as constipation and polypharmacy (Li et al. 2024).

Mechanistic insights are predominantly derived from transgenic and germ-free animal models, which have been instrumental in demonstrating potential links between microbial metabolites, neuroinflammation, and amyloid-related pathways. However, these models incompletely recapitulate the complexity and temporal progression of sporadic human Alzheimer’s disease, limiting direct translational inference. Importantly, evidence supporting a causal role of microbiota-derived signals in amyloid-β pathology in humans remains indirect, and longitudinal data assessing whether microbiota changes precede cognitive decline are scarce (Chandra et al. 2023).

Collectively, these findings suggest that gut microbiota alterations in AD are best interpreted as components of a multifactorial disease network rather than isolated drivers of pathology. Future studies integrating longitudinal sampling, host genetic and epigenetic profiling, and standardized microbiome methodologies will be essential to clarify causality and identify patient subgroups most likely to benefit from microbiome-targeted interventions. In AD, microbiota alterations are most often discussed as modulators of inflammatory and metabolic pathways that may influence amyloid- and tau-related processes, particularly during early-to-mid disease phases. From a genetic and epigenetic perspective, host variability in immune-related and metabolic regulatory pathways may modulate how microbiota-derived signals influence amyloid-related processes. Epigenetic mechanisms, including histone modifications and DNA methylation changes induced by microbial metabolites, may therefore act as intermediaries linking genetic susceptibility to inflammation-driven amyloid pathology in Alzheimer’s disease (Woo and Alenghat 2022).

### Parkinson’s disease

Beyond AD, increasing evidence also implicates the GM in Parkinson’s disease (PD). The observation that gastrointestinal symptoms precede motor dysfunction supports the hypothesis that PD pathology may originate in the gut (Sun et al. 2021). Studies have documented reduced microbial diversity in PD patients, characterized by decreased anti-inflammatory taxa and increased pro-inflammatory species (Aho et al. 2019; Hill-Burns et al. 2017; Hirschberg et al. 2019; Scheperjans et al. 2015). Microbiota-derived endotoxins and inflammatory signals may propagate to the brain via the vagus nerve, leading to dopaminergic neuronal injury. Experimental data support this, as FMT from PD patients into mice induced motor impairments and neuronal loss, whereas FMT from healthy donors maintained motor function (Nohesara et al. 2023; Sampson et al. 2016; Sun et al. 2021). Moreover, antibiotic treatment in α-synuclein-overexpressing mice improved motor performance and reduced neurodegeneration in the substantia nigra (Wei et al. 2022). These findings underscore the contribution of GM alterations to PD pathogenesis and the progression of neurodegeneration (Sun et al. 2021; Wei et al. 2022).

Multiple primary human studies have consistently reported PD-associated microbiome alterations, supporting a robust clinical signal compared with several other neurodegenerative disorders. Early case–control work linked the relative abundance of Enterobacteriaceae to postural instability and gait difficulty, suggesting an association between gut microbial composition and motor phenotype. Subsequent cohort studies further indicated that PD status and medication exposure show distinct microbial signatures, emphasizing the need to account for pharmacological and gastrointestinal confounders in microbiome analyses. In addition, metagenomic investigations in early-stage or medication-naïve PD have reported functional shifts related to microbial metabolism and barrier/immune interactions, strengthening the plausibility of microbiota-driven mechanisms in PD pathophysiology (Bedarf et al. 2017).

The association between gut microbiota alterations and Parkinson’s disease is supported by a substantial and growing body of evidence; however, important limitations constrain the interpretation of these findings. Several studies consistently report reduced levels of short-chain fatty acid–producing bacteria and increased abundance of pro-inflammatory taxa in PD patients, yet the specific microbial signatures vary considerably across cohorts. Differences in disease duration, motor versus non-motor symptom dominance, dietary patterns, and geographical background likely contribute to this variability. Moreover, gastrointestinal dysfunction, particularly constipation, represents a major confounding factor that is inconsistently controlled across studies (Nishiwaki et al. 2020).

Mechanistic hypotheses linking gut dysbiosis to α-synuclein aggregation and neuroinflammation are largely derived from animal models, including α-synuclein–overexpressing mice and fecal microbiota transplantation experiments (Sampson et al. 2016). While these models provide compelling support for a gut–brain signaling axis, they cannot fully capture the heterogeneity and multifactorial nature of idiopathic Parkinson’s disease in humans. In addition, the widespread use of dopaminergic and anticholinergic medications further complicates causal inference, as these treatments themselves can alter gut microbial composition. Importantly, whether microbiota alterations represent an initiating factor in PD pathogenesis or arise as a consequence of prodromal autonomic dysfunction remains unresolved. Longitudinal studies in at-risk populations, combined with integrative analyses incorporating host genetics, epigenetic regulation, and microbial metabolomics, will be critical to disentangle causality and identify mechanistically relevant microbiome-based therapeutic targets. Notably, in PD, microbiota-related signals may be particularly relevant at prodromal stages, given early gastrointestinal dysfunction and autonomic alterations. Importantly, genetic susceptibility affecting immune regulation and lysosomal function may shape individual responses to microbiota-derived inflammatory and metabolic cues in Parkinson’s disease. Microbiota-driven epigenetic modulation of genes involved in neuroinflammation and α-synuclein handling may therefore contribute to interindividual variability in disease onset, progression, and response to gut-targeted interventions (Woo and Alenghat 2022).

### Huntington’s disease

The gut–brain connection also warrants attention in Huntington’s disease (HD) and other genetically determined neurodegenerative disorders, where emerging evidence suggests that microbiota-driven mechanisms may intersect with host genetic vulnerabilities (Ekwudo et al. 2025).

In transgenic Huntington’s disease (HD) mouse models, sex-specific microbiota alterations have been observed. Male HD mice exhibited increased levels of Bacteroidetes and Lactobacillus with reduced abundance of Clostridium species, whereas female mice demonstrated elevations in taxa such as Bacteroidales and Burkholderiales. In human HD patients, fecal microbiota analyses revealed significant reductions in beneficial taxa such as Clostridium XVIII compared to healthy controls, suggesting that these shifts may influence immune regulation. Such findings indicate that specific imbalances within the GM may affect both motor function and behavioral processes (Fu et al. 2024). Notably, in HD models, microbial alterations have been shown to exacerbate behavioral abnormalities, increase intestinal permeability, and promote inflammatory responses. Experimental studies in diseased mice demonstrated that the intestinal barrier becomes more permeable relative to healthy controls, thereby aggravating neuroinflammation (Fu et al. 2024).

Compared with other neurodegenerative disorders, evidence linking gut microbiota alterations to Huntington’s disease remains limited and largely exploratory. Existing studies, predominantly derived from transgenic animal models, suggest that mutant huntingtin–driven cellular vulnerability may interact with microbiota-induced metabolic and inflammatory stressors. However, human data are sparse, often derived from small cohorts, and lack longitudinal follow-up, limiting robust conclusions regarding disease-specific microbial signatures (Wasser et al. 2020a, b).

Animal studies have provided valuable insights into potential links between gut dysbiosis, metabolic dysfunction, and neuroinflammation in Huntington’s disease. Nevertheless, these models represent a monogenic condition with relatively predictable onset, which differs fundamentally from the complex gene–environment interactions observed in other neurodegenerative diseases. Moreover, the extent to which microbiota alterations observed in animal models reflect primary disease mechanisms or secondary consequences of weight loss, dietary changes, and reduced mobility in advanced disease stages remains unclear (van der Burg et al. 2011).

At present, gut microbiota changes in Huntington’s disease should be interpreted cautiously as potential modifiers of disease expression rather than established contributors to pathogenesis. Future studies integrating microbiome profiling with metabolic, epigenetic, and clinical parameters in well-characterized patient cohorts will be essential to define the translational relevance of microbiome-based interventions in this disorder. Although Huntington’s disease is primarily driven by a monogenic mutation, genetic background and epigenetic plasticity may modulate vulnerability to microbiota-induced metabolic and inflammatory stress. In this context, microbiota-related epigenetic effects are more likely to act as disease modifiers rather than primary drivers of pathology (Gatto et al. 2020).

### Multiple sclerosis

Multiple sclerosis (MS), an autoimmune disorder, represents one of the most extensively studied conditions in relation to the GM. Patients with MS consistently display elevated levels of Akkermansia muciniphila and Methanobrevibacter smithii (Hirschberg et al. 2019). These bacteria are thought to trigger immune-activating pathways that contribute to disease relapses. In mouse models of MS, suppression of the GM through antibiotic treatment significantly reduced disease severity. Broad-spectrum antibiotics in particular were found to decrease pro-inflammatory T helper 17 (Th17) cell populations, thereby ameliorating symptoms in the experimental autoimmune encephalomyelitis (EAE) model. Furthermore, Bacteroides fragilis has been shown to produce capsular polysaccharide A (PSA), which markedly alleviated MS-like symptoms in mice while modulating immune responses. Collectively, these findings underscore the role of the gut flora in MS pathogenesis (Hirschberg et al. 2019).

Evidence linking gut microbiota dysbiosis to multiple sclerosis is comparatively stronger than for other neurodegenerative disorders, particularly with respect to immune-mediated mechanisms. Several studies have consistently identified alterations in microbial taxa associated with pro-inflammatory Th17 responses and reduced regulatory T cell–promoting bacteria in MS patients. Nonetheless, reported microbial signatures vary across cohorts, reflecting differences in disease subtype, treatment status, geographic location, and sequencing methodology. Importantly, many studies include patients receiving immunomodulatory therapies, which can independently influence gut microbial composition and confound disease-specific interpretations (Jangi et al. 2016).

Experimental autoimmune encephalomyelitis models have been instrumental in demonstrating that microbiota manipulation can alter disease severity and immune polarization. However, these models represent an induced inflammatory condition and do not fully recapitulate the chronic, heterogeneous, and relapsing nature of human multiple sclerosis. As a result, while animal studies strongly support a modulatory role of the microbiome in immune regulation, direct extrapolation to human disease progression remains limited (Constantinescu et al. 2011).

Collectively, current evidence suggests that gut microbiota alterations in MS function primarily as disease-modifying factors rather than sole drivers of pathology. Integrative longitudinal studies incorporating microbiome profiling, immune phenotyping, and epigenetic regulation are required to determine whether specific microbial patterns can predict disease activity, treatment response, or long-term neurological outcomes. In MS, microbiota effects are primarily interpreted as immune-modifying drivers that shape relapse activity and inflammatory burden. In the context of multiple sclerosis, host genetic variation influencing immune cell differentiation and tolerance may determine how gut microbiota alterations translate into pathogenic or protective immune responses. Epigenetic regulation of immune-related genes, modulated by microbiota-derived metabolites, provides a mechanistic link between genetic predisposition, microbial signals, and disease activity (Duarte-Silva et al. 2022).

### Shared mechanisms and therapeutic perspectives

A unifying mechanism across these disorders appears to involve microbiota-derived signals modulating CNS activity and immune regulation. Reduced SCFA production, accumulation of harmful microbial metabolites, and inflammatory signaling transmitted via the vagus nerve converge on microglial activation and neuroinflammation within the CNS (Mu et al. 2016). Improved understanding of these microbiota–CNS interactions may aid in the development of early diagnostic markers and therapeutic interventions for NDs (Chen et al. 2025).

Clinical and preclinical evidence supports this therapeutic potential. In a randomized controlled trial involving 60 AD patients, daily supplementation with fermented milk products containing Lactobacillus and Bifidobacterium strains for 12 weeks significantly improved Mini-Mental State Examination (MMSE) scores. These patients also exhibited reductions in inflammatory markers and increases in anti-inflammatory bacterial taxa (Akbari et al. 2016). Animal studies further corroborate these findings, showing that probiotic supplementation reduces Aβ plaque deposition and improves cognitive performance (Alatan et al. 2024; Athari Nik Azm et al. 2018).

Strategies targeting the microbiota—including prebiotics, probiotics, synbiotics, and fecal microbiota transplantation (FMT)—are increasingly recognized as promising approaches in both experimental and clinical research (Sun et al. 2021). In conclusion, the interaction between the GBA and the microbiota should be considered not merely as a consequence of NDs but also as a contributing causal factor. Accordingly, the microbiota warrants attention both as a therapeutic target and as a biomarker, with deeper investigations likely to yield more effective treatment strategies (Chen et al. 2025a, b). A summary of microbiota alterations associated with major neurodegenerative diseases is provided in Table 1. Key human cohort findings supporting these patterns are also discussed within the disease-specific sections, with clinical versus preclinical evidence explicitly distinguished.

**Table 1:** Microbiota alterations in major neurodegenerative diseases

Disease	Microbiota Changes	Functional Consequences	Study Type	Key evidence/Notes	References
Alzheimer’s disease (AD)	↓Firmicutes, ↑Bacteroidetes; ↓SCFA-producing bacteria (e.g., *Faecalibacterium*); ↑pro-inflammatory species	Increased gut permeability, Aβ accumulation, neuroinflammation	Clinical + animal	Human observational studies and transgenic mouse models; strong confounding by age, diet, and medication; predominantly associative evidence	(Sun et al. 2021; Zhu et al. 2022)
Parkinson’s disease (PD)	↓*Prevotella*; ↑Enterobacteriaceae; ↓anti-inflammatory bacteria	Reduced SCFA production, increased intestinal permeability, α-synuclein aggregation, dopaminergic neuron loss	Clinical + animal	Human cohort studies and α-synuclein–based animal models; early gastrointestinal dysfunction and constipation as major confounders; causality remains unresolved	(Hirschberg et al. 2019; Wei et al. 2022)
Huntington’s disease (HD)	↓*Clostridium XVIII*; altered *Lactobacillus*; ↑Bacteroidales, Burkholderiales (sex-specific patterns)	Increased gut permeability, immune dysregulation, behavioral and motor dysfunction	Animal + limited clinical	Predominantly transgenic animal models; limited human data; microbiota changes may reflect metabolic and disease-stage effects	(Fu et al. 2024; Gubert et al. 2020; Kong et al. 2020)
Multiple sclerosis (MS)	↑*Akkermansia muciniphila*, ↑*Methanobrevibacter smithii*; ↓*Bacteroides fragilis*	Altered Th17/Treg balance, enhanced pro-inflammatory signaling, demyelination	Clinical + animal	Human studies and EAE models; strong immune-mediated mechanisms; microbiota effects may be modified by immunomodulatory therapies	(Cuevas-Sierra et al. 2019; Hirschberg et al. 2019)

## Potential therapeutic approaches

Although no definitive cure currently exists for NDs, therapeutic strategies targeting the GM have increasingly gained attention in recent years (Sun et al. 2021). Through its bidirectional communication with the central nervous system (CNS), the GM regulates key processes including metabolism, inflammation, neurotransmitter production, and immune responses (Quigley 2017). Thus, modulation of the microbiota is considered a promising strategy that may reduce disease progression and significantly alleviate symptom severity (Hirschberg et al. 2019).

**Fig. 3:**
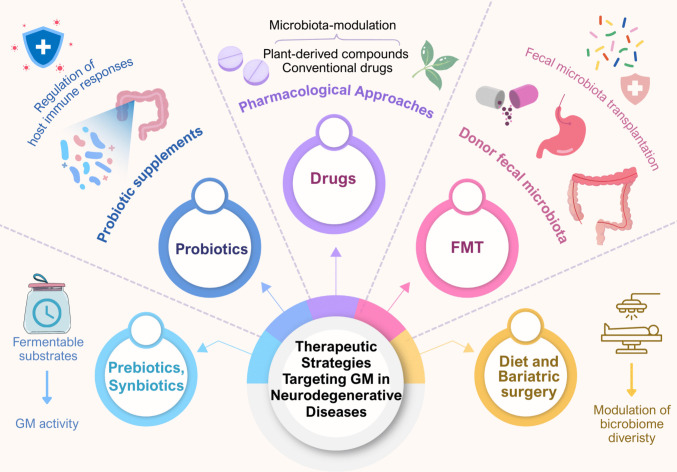
Therapeutic strategies targeting the gut microbiota in neurodegenerative diseases

Potential interventions include dietary modifications, bariatric surgery, probiotics, prebiotics, synbiotics, pharmacological agents, and fecal microbiota transplantation (FMT) (Ihlamur et al. 2024; Sun et al. 2021). These approaches primarily aim to enrich beneficial microbial species, enhance microbial diversity, reduce pathogenic taxa, and suppress inflammation (Zhu et al. 2022). Both clinical studies and experimental models have demonstrated that such interventions can induce meaningful alterations within the gut–brain axis (GBA), improving cognitive function, motor performance, and neuroinflammatory outcomes (Fu et al. 2024). As illustrated in Fig. 3, microbiota-targeted interventions may converge on shared metabolic and epigenetic pathways while exerting disease- and patient-specific effects.

Vagus nerve stimulation (VNS), including non-invasive modalities such as transcutaneous auricular VNS, has emerged as a promising approach to modulate the neural arm of the gut–brain axis. Beyond its established effects on autonomic regulation and central neurocircuitry, accumulating evidence suggests that vagal stimulation can influence intestinal barrier function, immune tone, and systemic inflammatory signaling through activation of the cholinergic anti-inflammatory pathway. Importantly, experimental and early clinical studies indicate that neuromodulation via the vagus nerve may also indirectly affect gut microbial composition and diversity by reshaping immune–metabolic interactions within the intestinal environment (Zhang et al. 2025).

Although direct evidence for microbiota-mediated effects of VNS in neurodegenerative diseases remains limited, this approach highlights a mechanistically plausible strategy to target gut–brain communication without directly manipulating microbial communities. At present, VNS should be regarded as an emerging adjunct intervention whose efficacy and microbiome-related effects require validation in disease-specific, longitudinal trials incorporating integrated microbiome and metabolomic endpoints (Faraji et al. 2025).

The figure summarizes major approaches used to modulate the gut microbiota in the context of neurodegenerative diseases. These include probiotics, which regulate host immune responses and enhance beneficial microbial taxa; prebiotics and synbiotics, which provide fermentable substrates and support gut microbiota activity; pharmacological approaches, where conventional drugs and plant-derived compounds exert microbiota-modulating effects; fecal microbiota transplantation (FMT), which introduces donor microbiota to re-establish microbial balance; and diet-induced weight loss and bariatric surgery, which reshape microbiome diversity and metabolite production. Together, these interventions aim to improve gastrointestinal and neurological outcomes by restoring a healthier microbial ecosystem.

### Diet-ınduced weight loss and bariatric surgery

Calorie-restricted diets and physical exercise have been shown to beneficially modulate the GM, leading to reductions in Enterobacteriaceae and sulfate-reducing bacteria, along with increases in the Bacteroides–Prevotella group. However, individuals with low microbial gene richness exhibit weaker responses and more pronounced inflammatory reactions under these interventions (Ahmad et al. 2023).

Bariatric surgical procedures, such as Roux-en-Y gastric bypass (RYGB), induce weight loss by altering microbial composition, including increases in Gammaproteobacteria, reductions in methanogens and Firmicutes, and enrichment of Faecalibacterium prausnitzii. These shifts are accompanied by elevated production of GLP-1, PYY, and GABA, which collectively support appetite regulation and improved insulin sensitivity. Nevertheless, RYGB has also been associated with reductions in beneficial taxa such as Bifidobacteria and Lactobacillus. Thus, while dietary and surgical interventions confer beneficial effects, they may also introduce unfavorable microbial changes. Despite these limitations, both strategies remain valuable for enhancing microbial diversity and improving host metabolic health (Hernández-Montoliu et al. 2023).

### Probiotics

Probiotics are defined as live microorganisms that, when administered in adequate amounts, confer health benefits to the host by modulating the GM (Celiker and Kalkan 2020). These organisms enhance the abundance of beneficial bacterial populations, suppress the proliferation of pathogenic taxa, and regulate host immune responses (Celiker and Kalkan 2020; Sun et al. 2021). Through the production of SCFAs, immunomodulatory compounds, and neurotransmitters, probiotics influence central nervous system (CNS) function via the gut–brain axis (GBA) (Sun et al. 2021; Wei et al. 2022).

Animal studies have demonstrated that strains such as Lactobacillus casei, Bifidobacterium bifidum, and Bifidobacterium lactis reduce oxidative stress and attenuate inflammation (Sun et al. 2021). In APP/PS1 transgenic mice, probiotic supplementation significantly reduced Aβ plaque deposition, strengthened the intestinal barrier, and enhanced SCFA production (Wei et al. 2022). Similar outcomes have been reported in clinical trials, where probiotic treatment generally improved the overall condition of patients with Alzheimer’s disease (AD). However, in advanced-stage AD patients, some trials reported no functionally significant effects (Sun et al. 2021). These findings suggest that the efficacy of probiotics depends on multiple factors, including the strain administered, dosage, and stage of disease.

### Prebiotics and synbiotics

Prebiotics are fermentable substrates that selectively modulate the composition and activity of the gastrointestinal microbiota in ways beneficial to the host. Resistant to digestive enzymes and gastric acidity, they reach the colon intact, where they undergo fermentation and promote the growth of beneficial bacteria. Common prebiotics include soybeans, lignin, oligosaccharides, and chicory root. By stimulating SCFA production, prebiotics reinforce the intestinal mucosal barrier, enhance immune function, and inhibit pathogenic colonization (Davani-Davari et al. 2019; Gibson et al. 2017).

Synbiotics, defined as formulations combining prebiotics and probiotics, aim to prolong probiotic survival, increase their functional activity, and establish more stable effects in the gut. Synbiotic consumption has been studied in the context of inflammatory bowel disease (IBD), type 2 diabetes (T2D), and colorectal cancer. For example, synbiotics enriched with inulin have been reported to alleviate ulcerative colitis symptoms, while the combined administration of butyrate and inulin improved metabolic outcomes by lowering fasting glucose levels in individuals with T2D. Synbiotics may also exert immunoregulatory effects by modulating apoptosis pathways, thereby reducing disease risk (Al-Habsi et al. 2024; Roshanravan et al. 2017).

Clinical studies have shown that fructooligosaccharides (FOS) resist digestion in the upper GIT, reach the colon intact, and, when metabolized together with probiotics, enhance the growth of beneficial bacterial taxa. Therefore, both prebiotic and synbiotic formulations should be considered as important modulators of microbial balance in healthy individuals and patients alike (Celiker and Kalkan 2020).

### Pharmacological approaches

Interindividual variability in drug responses has been widely documented, and growing evidence suggests that the GM plays an important role in shaping these outcomes. For example, a significant association has been identified between secondary bile acids—gut-derived metabolites—and the variability in patient responses to simvastatin. Similarly, bacterial production of p-cresol has been shown to influence host capacity for paracetamol sulfonation (Clayton et al. 2009).

Some drugs exert therapeutic effects in part by directly modulating the GM. In neonatal non-obese diabetic (NOD) mice, treatment with the glycopeptide antimicrobial agent vancomycin delayed diabetes onset and increased the abundance of Akkermansia muciniphila. Likewise, metformin, a first-line treatment for type 2 diabetes (T2D), improved metabolic dysfunction and reduced adipose tissue inflammation in high-fat diet-fed mice, while simultaneously promoting mucin production in goblet cells and increasing Akkermansia muciniphila abundance (Hansen et al. 2012; Shin et al. 2014).

Beyond conventional drugs, plant-derived compounds also exhibit microbiota-modulating effects. Berberine, isolated from Coptis chinensis, increased SCFA production and normalized the Bacteroidetes/Firmicutes ratio in high-fat diet-induced obesity models. Collectively, these findings demonstrate that the GM can significantly shape both drug efficacy and side-effect profiles, highlighting the importance of considering host–microbiota interactions in pharmacological interventions (Zhao et al. 2023).

### Fecal microbiota transplantation

Fecal microbiota transplantation (FMT) involves the transfer of fecal microbiota from healthy donors to patients via oral capsules, upper gastrointestinal intubation, endoscopy, or rectal enema. Currently, FMT is an established therapy for recurrent Clostridioides difficile infection (CDI) and is considered one of the most effective interventions for restoring gut microbial balance (Wei et al. 2022).

Emerging clinical evidence suggests that FMT may also provide neurological benefits. For instance, in a male patient with concurrent CDI and Alzheimer’s disease (AD), FMT not only resolved the infection but also led to marked cognitive improvement (Sun et al. 2021). Similarly, in a study of six male PD patients, five showed significant improvements in both motor and non-motor symptoms within six weeks post-FMT, with only one reporting mild adverse effects (Wei et al. 2022).

Animal studies further elucidate the therapeutic potential of FMT in NDs models. In AD models, transplantation of microbiota from healthy donors increased SCFA production, reduced phosphorylated tau (pTau) and Aβ plaque accumulation, attenuated inflammation, improved cognitive performance, and enhanced synaptic plasticity (Sun et al. 2021). For example, in the ADLPAPT transgenic mouse model, FMT from healthy donors strengthened the intestinal barrier, reduced Aβ deposition, suppressed glial activation, and improved spatial learning and memory. Conversely, transplantation of microbiota from AD patients into APP/PS1 transgenic mice upregulated NLRP3 inflammasome expression, induced inflammation, increased microglial activation, and exacerbated cognitive decline—effects that were reversed by healthy donor FMT (Kim et al. 2020; Shen et al. 2020).

In PD models, FMT restored GM composition, reduced lipopolysaccharide (LPS) levels in the colon and serum, inhibited the TLR4/PI3K/AKT/NF-κB inflammatory signaling pathway, preserved both BBB and intestinal integrity, decreased systemic inflammation, and exerted neuroprotective effects (Wei et al. 2022).

Taken together, these findings demonstrate that FMT offers a rapid, cost-effective, and feasible strategy for GM modulation, with strong potential as a selective and effective therapeutic approach in NDs (Sun et al. 2021; Wei et al. 2022). An overview of therapeutic strategies targeting the GM and their evidence in NDs is summarized in Table 2. The table includes both microbiota-directed and gut–brain axis–oriented interventions, with study type and translational limitations indicated.

**Table 2:** Therapeutic strategies targeting gut microbiota in neurodegenerative diseases

Strategy	Mechanism of Action	Evidence in NDs	Study Type	Limitations	References
Probiotics	↑SCFA production, ↓inflammation, modulation of neurotransmitters	Improved cognition in AD patients, ↓Aβ plaques in AD mice	Clinical + animal	Effects strain- and dose-dependent; inconsistent results in late-stage AD	(Akbari et al. 2016; Sun et al. 2021; Wei et al. 2022)
Prebiotics	Selectively stimulate beneficial bacteria, enhance SCFA	Improved barrier integrity; reduced inflammation in metabolic and gut disorders	Animal + indirect clinical	Few NDs-specific clinical trials	(Celiker and Kalkan 2020)
Synbiotics	Combination of probiotics + prebiotics; enhanced colonization	Reduced inflammation; improved metabolic outcomes in animal studies	Animal + limited clinical	Limited NDs studies	(Celiker and Kalkan 2020)
FMT	Reset microbiota composition by transplanting donor microbiome	Improved cognition and motor functions in AD and PD models; case reports of clinical improvement	Animal + case reports	Safety concerns, donor variability	(Sun et al. 2021; Wei et al. 2022)
Diet & Bariatric surgery	Modulate microbiome diversity, metabolites (GLP-1, SCFA)	Improved insulin sensitivity, reduced inflammation; potential indirect NDs benefits	Clinical + animal	Mixed long-term effects; reduced *Bifidobacteria* and *Lactobacillus* post-surgery	(Hirschberg et al. 2019)
Vagus nerve stimulation (VNS/taVNS)	Neural modulation of the gut–brain axis; activation of the cholinergic anti-inflammatory pathway; indirect regulation of immune tone, intestinal barrier function, and gut microbial diversity	Emerging/early-stage evidence	Preclinical + early clinical (indirect ND evidence)	Limited ND-specific trials; heterogeneity in stimulation parameters; lack of integrated microbiome endpoints	(Breit et al. 2018; Liu et al. 2024)

## Clinical ımplications and translational perspective

The accumulating evidence linking GM dysbiosis to NDs has important clinical and translational implications. First, microbiota-derived signatures—such as reduced short-chain fatty acid (SCFA) levels, increased intestinal permeability, and specific taxonomic shifts—may serve as early, minimally invasive biomarkers of disease risk and progression. Fecal, blood, or saliva-based microbiome and metabolite profiles could be integrated into screening algorithms to complement genetic and neuroimaging markers in Alzheimer’s disease (AD), Parkinson’s disease (PD), Huntington’s disease (HD), and Multiple sclerosis (MS).

Second, microbiota-targeted therapies have the potential to modify disease course rather than merely alleviate symptoms. Probiotics, prebiotics, synbiotics, diet-based interventions, and fecal microbiota transplantation (FMT) have been shown to restore microbial diversity, enhance SCFA production, and reduce neuroinflammation in preclinical models. Early clinical trials in AD and PD suggest improvements in cognitive and motor outcomes in selected patient subgroups. In the future, such interventions may be combined with disease-modifying drugs to create multimodal treatment regimens tailored to the individual patient’s microbiome profile.

Third, a better understanding of host–microbiota interactions is essential for precision medicine in NDs. Genetic polymorphisms in immune-related and barrier-regulating genes influence microbiota composition, while microbiota-derived metabolites modulate epigenetic processes in neuronal and immune cells. This bidirectional relationship implies that patients with similar clinical phenotypes may respond differently to microbiota-targeted therapies depending on their genetic and epigenetic background. Integrating microbiome data with host genomics, epigenomics, and clinical metrics using advanced bioinformatics and machine learning approaches may enable personalized risk prediction and therapeutic stratification.

Finally, translation into routine clinical practice will require standardized protocols for sample collection, microbiota analysis, and therapeutic interventions, as well as robust regulatory frameworks to ensure safety and quality control. Well-designed, multicenter randomized controlled trials are needed to determine optimal microbial strains, dosages, treatment durations, and patient selection criteria. Despite these challenges, the GM represents a promising and tractable target for innovative, patient-centered approaches to the prevention and management of neurodegenerative diseases.

### Translational limitations of animal and preclinical microbiome studies in neurodegeneration

Animal and preclinical models have played a central role in advancing mechanistic understanding of gut microbiota–brain interactions in neurodegenerative diseases. Germ-free mice, transgenic models, and fecal microbiota transplantation experiments have provided valuable insights into how microbial signals may influence neuroinflammation, protein aggregation, and neuronal vulnerability. However, important translational limitations must be considered when extrapolating these findings to human disease (Cryan et al. 2019).

Germ-free animals exhibit profound alterations in immune maturation, neurodevelopment, and metabolic regulation, resulting in phenotypes that do not accurately reflect physiological human conditions. Similarly, transgenic models of neurodegeneration typically capture only selected pathological features, such as amyloid or α-synuclein accumulation, while failing to reproduce the full clinical heterogeneity, comorbidity burden, and age-related complexity of sporadic human disease. As a result, microbiota-dependent effects observed in these models may overestimate causal relationships that are more nuanced in human populations (Jucker 2010).

Fecal microbiota transplantation studies further introduce variability related to donor selection, antibiotic pretreatment, housing conditions, and dietary standardization, all of which can strongly influence experimental outcomes. In addition, the controlled environments of animal studies contrast sharply with human settings, where diet, medication use, lifestyle factors, and socioeconomic variables exert substantial and often interacting effects on gut microbial composition (Rothschild et al. 2018).

In clinical contexts, neurodegenerative diseases are frequently accompanied by gastrointestinal dysfunction, polypharmacy, and systemic comorbidities that independently shape the gut microbiome. These factors complicate causal inference and limit the direct applicability of preclinical findings. Consequently, microbiota alterations observed in animal models should be interpreted as contributors to mechanistic plausibility rather than definitive evidence of disease causation (Suez et al. 2019).

Bridging the translational gap will require well-designed longitudinal human studies integrating microbiome profiling with host genetic susceptibility, epigenetic regulation, and detailed clinical phenotyping. Such integrative approaches are essential to determine whether microbiota changes precede disease onset, modify progression, or represent secondary adaptations to neurodegenerative pathology.

## Discussion

The link between NDs and the GM has recently become a central focus of both basic and clinical research. The literature reviewed in this article demonstrates that functional and compositional alterations within the GM contribute to NDs pathogenesis through effects on immune, central nervous system (CNS), and epigenetic processes (Hirschberg et al. 2019; Nohesara et al. 2023). At the core of these interactions lies the gut–brain axis (GBA), which mediates bidirectional communication between the digestive and nervous systems through immunological, molecular, and neuroendocrine pathways (Carabotti et al. 2015).

Findings are particularly compelling for Alzheimer’s disease (AD), where the association between microbiota composition and disease progression is most clearly established. AD pathology is characterized by amyloid-β (Aβ) plaque accumulation, tau hyperphosphorylation, and chronic neuroinflammation. Dysbiosis, marked by reductions in beneficial metabolite-producing bacteria, enrichment of proinflammatory taxa, and compromised intestinal barrier integrity, appears to exacerbate these processes. Animal studies in AD models have demonstrated that experimental manipulation of the GM can improve cognitive performance (Sun et al. 2021; Zhu et al. 2022), highlighting the direct impact of gut-derived signals on protein aggregation and inflammatory pathways in the CNS.

Epigenetic mechanisms provide an additional explanatory link between the GM and AD pathogenesis. Experimental findings indicate that microbial metabolites can influence chromatin-regulating processes such as histone acetylation and deacetylation. For instance, administration of histone deacetylase (HDAC) inhibitors has been shown to reduce plaque deposition and attenuate neuroinflammation in AD models. These observations suggest that microbiota-derived metabolites, such as SCFAs, may regulate gene expression at the epigenetic level and thereby modulate neurodegenerative processes (Stein and Riber 2023; Zhang and Schluesener 2013).

In Parkinson’s disease (PD), the presence of gastrointestinal symptoms preceding motor deficits strengthens the hypothesis that pathogenesis may be initiated in the gut (Fu et al. 2024). Microbiota analyses in PD patients commonly reveal reductions in Prevotella and increases in Enterobacteriaceae, changes that decrease SCFA production and compromise intestinal barrier integrity, thereby facilitating α-synuclein aggregation. Gut-derived inflammatory signals transmitted via the vagus nerve to the CNS may represent an early trigger of dopaminergic neuronal loss. These mechanisms resemble those observed in AD, particularly in relation to inflammation and barrier dysfunction, though they diverge at the molecular level of disease pathophysiology (Liu et al. 2020; Yang et al. 2022).

Data on Huntington’s disease (HD) are more limited, but existing evidence suggests that reduced microbial diversity and diminished production of neuroprotective metabolites may contribute to disease progression. Associations between microbial imbalances and the severity of cognitive and motor impairments strengthen the role of the GBA in HD. Furthermore, disruptions in DNA methylation and histone acetylation at the epigenetic level are known to impair CNS gene expression. The beneficial effects of HDAC inhibitors in AD models raise the possibility that similar strategies could hold therapeutic promise in HD (Wasser et al. 2020a, b).

MS, an autoimmune-mediated NDs, represents another condition strongly linked to microbiota alterations. In MS, dysbiosis is frequently associated with increased abundance of Akkermansia muciniphila and reduced SCFA production (Cuevas-Sierra et al. 2019; Hirschberg et al. 2019). These changes weaken intestinal barrier function, facilitate translocation of proinflammatory cytokines into circulation, and disrupt the Th17/Treg balance, thereby promoting myelin-damaging inflammatory processes within the CNS. Although these mechanisms parallel the barrier dysfunction and inflammation observed in AD and PD, their immunological basis is distinct (Nouri et al. 2014).

Across all NDs reviewed, several convergent mechanisms emerge: increased proinflammatory cytokines, impaired intestinal barrier integrity, reduced levels of beneficial metabolites such as SCFAs, and epigenetic alterations. Particularly notable are the positive findings from APP transgenic mouse models of AD treated with HDAC inhibitors, which suggest that combining microbiota-targeted interventions with epigenetic modulators may provide innovative therapeutic strategies to slow disease progression. While similar mechanisms may operate in PD, HD, and MS, disease-specific pathophysiological pathways must be addressed (Munteanu et al. 2024; Zhang et al. 2024).

Although the findings presented here are largely consistent with the broader literature, methodological variability, sample size limitations, and population heterogeneity remain sources of discrepancy across studies. Some investigations report no microbiota differences in AD or PD patients, whereas others observe pronounced alterations—likely reflecting influences such as genetic predisposition, dietary habits, analytic methods, and disease stage at the time of sampling (Li et al. 2024).

In conclusion, this review highlights the pivotal role of the GM in NDs, integrating evidence from genetic and epigenetic perspectives. The microbiota–disease relationship is most strongly supported in AD, but growing evidence points to similar contributions in PD, HD, and MS. Future multicenter, longitudinal studies with standardized methodologies will be essential to establish the clinical utility of microbiota-based strategies in NDs management.

### Limitations

This review has several limitations that should be considered when interpreting the findings. First, the majority of available data on GM and NDs derive from cross-sectional or small-scale cohort studies, which limits causal inference. Differences in study design, sequencing platforms, bioinformatic pipelines, and statistical methods contribute to substantial heterogeneity across microbiome studies, making direct comparisons challenging and sometimes leading to inconsistent results.

Second, most mechanistic insights into microbiota–brain interactions stem from animal models, germ-free mice, or in vitro experiments. While these models provide valuable information about causal pathways, their translational relevance to human disease is not always straightforward, particularly in disorders with complex genetic and environmental backgrounds such as AD, PD, HD, and MS. Third, this work was conducted as a narrative review rather than a systematic review or meta-analysis. Although we applied explicit inclusion and exclusion criteria and focused on peer-reviewed literature, we did not perform a formal risk-of-bias assessment or quantitative synthesis. Therefore, publication bias and selective reporting may have influenced the evidence base.

Finally, the microbiota is highly sensitive to diet, lifestyle, medication use, geography, and host genetics. Many of the studies included here did not fully adjust for these confounding factors, which may have affected the reported associations between GM composition, epigenetic alterations, and ND outcomes. Future large-scale, longitudinal studies with standardized methodologies and comprehensive data collection will be essential to validate and refine the conclusions drawn in this review.

### Future directions

Future research on the gut–brain axis in neurodegenerative diseases should focus on several key areas:

- **Multi-omics approaches:** Integrating metagenomics, metabolomics, transcriptomics, and epigenomics to comprehensively characterize microbiota–host interactions.
- **Mechanistic epigenetic studies**: Exploring how microbiota-derived metabolites (e.g., SCFAs) regulate DNA methylation, histone modification, and non-coding RNAs in neuronal and immune cells.
- **Personalized medicine:** Developing patient-specific microbiota modulation strategies, considering inter-individual variability in microbiome composition.
- **Artificial intelligence and bioinformatics:** Applying machine learning to large-scale microbiome and epigenetic datasets for risk prediction and therapeutic stratification.
- **Clinical trials:** Conducting well-designed, multicenter, randomized controlled trials testing probiotics, prebiotics, synbiotics, and FMT in Alzheimer’s, Parkinson’s, Huntington’s disease, and MS.
- **Combination therapies:** Investigating synergistic effects of microbiota-targeted interventions and epigenetic modulators (e.g., HDAC inhibitors) to slow disease progression (Kaur et al., 2021); Loh et al. 2024).

## Conclusion and recommendations

In this review, the complex interactions between the GM and NDs were evaluated in the context of genetic and epigenetic mechanisms. The literature reviewed indicates that alterations in GM composition extend beyond the GIT, exerting direct effects on metabolic pathways, the immune system, and the central nervous system (CNS) (Hirschberg et al. 2019; Nohesara et al. 2023). Across multiple NDs—including Alzheimer’s disease (AD), Huntington’s disease (HD), Parkinson’s disease (PD), and MS—the gut–brain axis (GBA) has been shown to mediate pathological mechanisms with overlapping features. These include the enrichment of proinflammatory bacteria, disruption of intestinal barrier integrity, depletion of beneficial metabolites, and epigenetic alterations (Cuevas-Sierra et al. 2019; Sun et al. 2021).

In AD, dysbiosis has been strongly linked to amyloid plaque accumulation, tau hyperphosphorylation, cognitive decline, and neuroinflammation, with evidence from both clinical studies and experimental animal models (Zhu et al. 2022). Findings from APP transgenic mice demonstrate that HDAC inhibitor treatment significantly reduces plaque pathology and alleviates neuroinflammatory processes, supporting the role of microbiota-derived metabolites in shaping disease progression via epigenetic regulation. In PD, decreased Prevotella and increased Enterobacteriaceae reduce SCFA production, weaken intestinal barrier integrity, and accelerate α-synuclein aggregation (Fu et al. 2024; Sampson et al. 2016; Scheperjans et al. 2015). Similarly, reduced microbial diversity, impaired immune balance, and increased inflammatory responses have been reported in HD and MS. Collectively, these findings underscore the potential of microbiota-targeted and epigenetic-based therapeutic strategies in the prevention and management of NDs.

Based on the evidence synthesized, the following recommendations are proposed:

- **Large-scale and longitudinal studies:** Future research should involve larger patient cohorts with stratification by disease stage to better characterize the role of microbiota alterations in disease onset and progression (Hirschberg et al. 2019).
- **Methodological standardization:** Standardized protocols for sample collection, storage, sequencing, and data analysis are needed to improve comparability across studies (Nohesara et al. 2023).
- **Mechanistic epigenetic studies:** Comprehensive in vitro and in vivo investigations into how microbiota-derived metabolites modulate DNA methylation, histone acetylation, and other epigenetic processes will facilitate the development of targeted therapeutic strategies (Sun et al. 2021; Zhu et al. 2022).
- **Clinical ıntervention trials:** The efficacy of microbiota-targeted therapies—such as probiotics, prebiotics, synbiotics, and fecal microbiota transplantation (FMT)—should be further assessed through randomized controlled clinical trials in NDs populations (Cuevas-Sierra et al. 2019).
- **Personalized therapeutic approaches:** Given the variability of microbiota composition across individuals due to lifestyle, genetic, and environmental factors, patient-specific microbiota modulation strategies should be developed to optimize treatment efficacy (Fu et al. 2024).

In the coming years, more detailed investigations into GM–NDs interactions are expected. Multi-omics approaches—including metagenomics, epigenomics, and transcriptomics—will be critical for elucidating microbiota–epigenetic relationships and developing novel biomarkers for early diagnosis (Hirschberg et al. 2019; Sun et al. 2021). Moreover, the integration of artificial intelligence and bioinformatics will enable comprehensive analysis of microbiome and epigenetic datasets from large patient cohorts, supporting improved disease risk prediction and personalized treatment planning (Fu et al. 2024; Zhu et al. 2022).

Finally, the combined application of epigenetic modulators and microbiota-derived metabolites holds promise for translational therapeutic development. In the long term, the integration of personalized microbiota modulation into clinical practice represents a powerful strategy for the prevention and management of NDs (Cuevas-Sierra et al. 2019; Nohesara et al. 2023).

## Data Availability

No datasets were generated or analysed during the current study.

## Abbreviations

AD: Alzheimer’s disease
BBB: Blood–brain barrier
CNS: Central nervous system
CDI: Clostridioides difficile infection
EAE: Experimental autoimmune encephalomyelitis
ENS: Enteric nervous system
FMT: Fecal microbiota transplantation
GABA: Gamma-aminobutyric acid
GBA: Gut–brain axis
GIT: Gastrointestinal tract
GM: Gut microbiota
HD: Huntington’s disease
IBD: Inflammatory bowel disease
ISAPP: International scientific association for probiotics and prebiotics
LPS: Lipopolysaccharide
MMSE: Mini-mental state examination
MS: Multiple sclerosis
NDs: Neurodegenerative diseases
PD: Parkinson’s disease
PSA: Polysaccharide A
RYGB: Roux-en-Y gastric bypass
SCFAs: Short-chain fatty acids
T2D: Type 2 diabetes
Th17: T helper 17 (cell)
Treg: Regulatory T (cell)

## Declarations

### Generative AI and AI-assisted technologies in the writing process

During the preparation of this work, ChatGPT tools were used to improve readability. The authors reviewed and edited the content as needed and took full responsibility for the content of the publication. 
